# Comparative cardiovascular risks of canagliflozin and selective SGLT2 inhibitors in type 2 diabetes

**DOI:** 10.3389/fphar.2025.1686851

**Published:** 2025-10-20

**Authors:** Edy Kornelius, Shih-Chang Lo, Yi-Sun Yang, Yu-Hsun Wang, Chien-Ning Huang

**Affiliations:** ^1^ Chung Shan Medical University, School of Medicine, Taichung, Taiwan; ^2^ Chung Shan Medical University Hospital, Department of Internal Medicine, Division of Endocrinology and Metabolism, Taichung, Taiwan; ^3^ Chung Shan Medical University Hospital, Department of Medical Research, Taichung, Taiwan; ^4^ Chung Shan Medical University, Institute of Medicine, Taichung, Taiwan

**Keywords:** SGLT1, SGLT2, MACE, mortality, diabetes

## Abstract

**Aims:**

Dual inhibition of sodium-glucose cotransporter (SGLT) 1 and 2 with canagliflozin may offer additional metabolic effects beyond selective SGLT2 inhibition; however, its comparative cardiovascular associations remain uncertain. This study compared the risks of major adverse cardiovascular events (MACE) and all-cause mortality between canagliflozin and selective SGLT2 inhibitors in routine clinical practice.

**Methods and results:**

We conducted a retrospective cohort study using a multicenter electronic health record database including over 118 million patients. Adults with type 2 diabetes, no prior cardiovascular disease, and new use of an SGLT inhibitor between January 2016 and December 2023 were identified. After applying strict exclusion criteria and 1:1 propensity score matching, 24,078 patients (mean age, 57 years; 47% women) were included: 12,039 initiated canagliflozin and 12,039 initiated other SGLT2 inhibitors. The primary outcome was MACE (composite of myocardial infarction, stroke, or all-cause mortality). Compared with other SGLT2 inhibitors, canagliflozin was associated with higher risk of MACE (hazard ratio [HR], 1.23; 95% confidence interval [CI], 1.14–1.33) and all-cause mortality (HR, 1.49; 95% CI, 1.33–1.68). Hemorrhagic stroke risk was also elevated (HR, 1.35; 95% CI, 1.02–1.79), while risks of ischemic stroke and myocardial infarction were similar.

**Conclusion:**

In this large real-world cohort, patients initiating canagliflozin had higher observed event rates for a composite of myocardial infarction, stroke, or all-cause mortality compared with those initiating selective SGLT2 inhibitors. These associations should be interpreted as exploratory and hypothesis-generating, given the observational design and differences from randomized trial evidence. Further research is needed to clarify potential differences among SGLT2 inhibitors in routine practice.

## Introduction

Sodium-glucose cotransporter 2 (SGLT2) inhibitors have emerged as an essential therapy for type 2 diabetes mellitus (T2DM) due to their glycemic benefits and robust cardiovascular and renal protective effects. (Li et al., 2021; Zelniker et al., 2019) Large cardiovascular outcome trials have demonstrated that SGLT2 inhibitors significantly reduce risks of heart failure hospitalization, progression of kidney disease, and even major adverse cardiovascular events (MACE) and mortality in patients with T2DM. (Neal et al., 2017; Wiviott et al., 2019; Zinman et al., 2015; Bhatt et al., 2021a). These benefits have led to broad use of SGLT2 inhibitors in patients with and without prior cardiovascular disease.

SGLT2 inhibitors primarily act in the kidney to prevent glucose reabsorption, promoting glycosuria. In contrast, SGLT1 is expressed in the intestinal mucosa and in other tissues, including kidney and heart. (Rieg and Vallon, 2018). Canagliflozin is considered a dual SGLT1/2 inhibitor due to its additional inhibition of SGLT1. By inhibiting SGLT1, canagliflozin can delay intestinal glucose absorption and enhance incretin release (GLP-1/GIP), which might improve postprandial glycemic control. (Martinussen et al., 2020) It has been hypothesized that adding SGLT1 inhibition to SGLT2 inhibition could provide additional metabolic and possibly cardiovascular benefits. (Pitt et al., 2022). Recent randomized trials of the dual SGLT1/2 inhibitor sotagliflozin, SCORED and SOLOIST, demonstrated significant reductions in heart-failure outcomes and hinted at lower rates of ischemic events, including stroke, in high-risk patients with type 2 diabetes. (Aggarwal et al., 2025; Bhatt et al., 2021b). However, the net cardiovascular impact of dual SGLT1/2 inhibition compared to selective SGLT2 inhibition remains uncertain. Some preclinical data raise questions about SGLT1’s role in the heart and other organs, leaving it unclear whether its inhibition is beneficial or harmful in the long term. (Li et al., 2019; Seidelmann et al., 2018).

Given the expanding use of SGLT2 inhibitors and the partial SGLT1 inhibition of canagliflozin, it is important to understand comparative safety and effectiveness. This study was designed to compare the cardiovascular outcomes of canagliflozin versus other selective SGLT2 inhibitors in a real-world T2DM population without established cardiovascular disease at baseline. We focused on MACE and all-cause mortality, hypothesizing that canagliflozin would be at least non-inferior to other SGLT2 inhibitors in terms of cardiovascular risk.

## Methods

### Study design and data source

We performed a retrospective cohort study using the TrinetX, U.S. Collaborative Network, a large multi-center electronic health record (EHR) database encompassing over 118 million patients across the United States. This data network includes de-identified patient information from participating healthcare organizations, with comprehensive capture of diagnoses, prescriptions, procedures, and outcomes. The study was conducted in accordance with the Strengthening the Reporting of Observational Studies in Epidemiology (STROBE) guidelines. (von et al., 2007). This study was approved by the Institutional Review Board of Chung Shan Medical University Hospital, under the approval number CS2-24180.

### Cohort selection

We identified adults (age ≥18 years) with T2DM who were new users of an SGLT inhibitor between Jan 1, 2016 and Dec 31, 2023. New use was defined as a first prescription record for any canagliflozin or other SGLT2 inhibitor with no prior prescription for an SGLT inhibitor in the patient’s record. The exposure of interest was dual SGLT1/2 inhibitor use, defined as initiation of canagliflozin only. The comparison group was initiation of other selective SGLT2 inhibitor (empagliflozin, dapagliflozin, or ertugliflozin). Patients were categorized based on their first SGLT inhibitor prescription in the study period. We excluded any patients who had evidence of using a different SGLT2 inhibitor class agent during the baseline period or follow-up (to ensure distinct exposure groups). In other words, patients in the canagliflozin group must not have used any selective SGLT2 inhibitor, and *vice versa*.

### Exclusion criteria

To focus on primary prevention and avoid confounding by pre-existing cardiovascular conditions, we excluded patients with any history of major cardiovascular disease or other serious conditions prior to the index date (the date of first SGLT inhibitor prescription). Specifically, we excluded individuals with a documented history of type 1 diabetes or gestational diabetes, transient ischemic attack (TIA), ischemic heart disease (including coronary artery disease or myocardial infarction), cerebrovascular disease (stroke), atherosclerosis or peripheral arterial disease, and end-stage renal disease (ESRD) or dialysis. These conditions were identified via diagnosis codes in the patient’s record. Patients without at least 6 months of medical history in the database prior to the index date were also excluded to ensure adequate baseline data for covariate assessment. After applying these criteria, we obtained two groups: patients who initiated canagliflozin and those who initiated other SGLT2 inhibitors.

### Propensity score matching (PSM)

**FIGURE 1:**
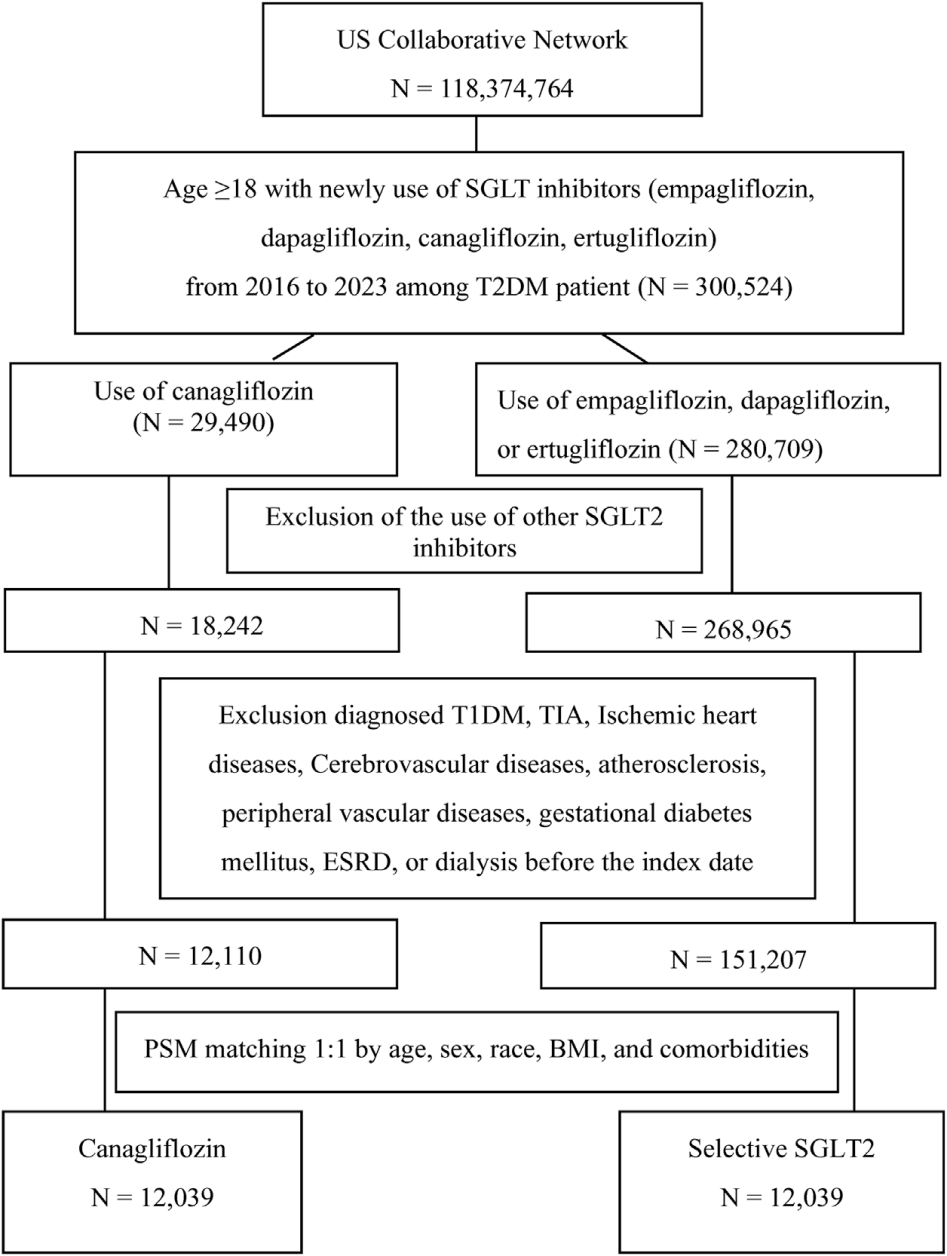
Flow diagram of this study.

Given the observational design, we used PSM to balance baseline characteristics between the two groups and mitigate confounding. The propensity score was estimated using a logistic regression model predicting the probability of receiving canagliflozin (versus other selective SGLT2 inhibitor) given baseline covariates. Covariates included demographics, baseline body mass index (BMI), baseline estimated glomerular filtration rate (eGFR), concurrent medications, and comorbidities. Each canagliflozin user was matched to one selective SGLT2 user with a similar propensity score using 1:1 nearest-neighbor matching without replacement (caliper = 0.1). Successful matching was confirmed by standardized mean differences (SMD) < 0.1 for all covariates, indicating negligible residual imbalance. After matching, the final analysis cohort consisted of 12,039 canagliflozin users and 12,039 other SGLT2 users. The cohort flow is illustrated in Figure 1, which depicts the initial population, application of inclusion/exclusion criteria, and the matching process leading to the final analytical sample.

### Exposure and follow-up

Exposure was defined at baseline by the category of SGLT inhibitor initiated. Patients were followed from the index date until the occurrence of an outcome event, discontinuation of the index drug, death, or end of the study period (whichever came first).

### Outcomes

The primary outcome was MACE, defined in this study as the first occurrence of myocardial infarction (MI), stroke (ischemic or hemorrhagic) or all-cause mortality. Secondary outcomes included individual outcome of all-cause mortality, MI alone, and stroke (all types). Each outcome was ascertained from diagnosis codes recorded during follow-up encounters or hospitalization records. Mortality was captured from discharge disposition data and EHR mortality indicators.

### Statistical analysis

We described baseline characteristics of the matched cohorts using means (standard deviation) for continuous variables and counts (percentages) for categorical variables. We used Kaplan-Meier curves to inspect event-free survival over time for the primary outcome. Cox proportional hazards regression was used to compare time-to-event outcomes between the canagliflozin group and the selective SGLT2 group in the matched sample, yielding hazard ratios (HRs) and 95% confidence intervals (CIs). For each outcome, an HR > 1.0 indicates higher risk with canagliflozin. We considered p < 0.05 (two-tailed) as statistically significant. Detailed coding of this study shown in Supplementary Table S1.

### Subgroup analyses

We conducted pre-specified subgroup analyses for the primary composite outcome and for all-cause mortality across key stratification variables: age (<65 vs. ≥65 years), sex (male vs. female), race (White, Black, Asian), BMI (<30 vs. ≥30), baseline eGFR (<60 vs. ≥60), and baseline HbA1c (<8.0% vs. ≥8.0%). For each subgroup, a separate Cox model was run on the matched subset, and the HR for dual vs. selective therapy was estimated. We assessed heterogeneity by examining whether the 95% CIs of different subgroups overlapped and by calculating interaction p-values. These subgroup analyses were exploratory to identify if certain patient characteristics modified the association between canagliflozin and outcomes. All analyses were conducted using the default analytic tools available within the TriNetX platform.

## Results

### Study population

After applying inclusion and exclusion criteria and propensity matching, we analyzed 24,078 patients with T2DM, with 12,039 in the canagliflozin group and 12,039 in the selective SGLT2 inhibitor group (Table 1). The two groups had a mean age of 57 years, and 47% were female in each, reflecting successful matching. Racial composition was also similar: approximately 61% White, 14% Black, 5% Asian, and the remainder of other/unknown races. Mean baseline HbA1c was 8.6% in both groups, indicating moderately uncontrolled diabetes on average. Comorbid conditions and medication use were well-balanced post-matching (all standardized differences <0.1). For example, the prevalence of hypertension (31%), hyperlipidemia (30.5%), and chronic kidney disease (3.3%).

**TABLE 1:** Demographic characteristics of canagliflozin and other selective SGLT2.

	Before PSM				After PSM			
	Canagliflozin N = 12110	Selective SGLT2 N = 151207	p	SMD	Canagliflozin N = 12039	Selective SGLT2 N = 12039	p	SMD
Age at Index	57.04 ± 12.03	58.10 ± 12.08	<0.001	0.088	57.02 ± 12.04	57.05 ± 11.26	0.858	0.002
Sex								
Female	5719 (47.23)	68563 (45.34)	<0.001	0.038	5677 (47.16)	5631 (46.77)	0.553	0.008
Male	5863 (48.42)	77113 (51.00)	<0.001	0.052	5836 (48.48)	5913 (49.12)	0.321	0.013
Race								
White	7391 (61.03)	86092 (56.94)	<0.001	0.083	7359 (61.13)	7337 (60.94)	0.771	0.004
Black or African American	1703 (14.06)	26157 (17.30)	<0.001	0.089	1700 (14.12)	1665 (13.83)	0.515	0.008
Asian	630 (5.20)	10747 (7.11)	<0.001	0.079	629 (5.23)	665 (5.52)	0.304	0.013
Other Race	690 (5.70)	8478 (5.61)	0.676	0.004	669 (5.56)	682 (5.67)	0.716	0.005
Unknown Race	1507 (12.44)	17368 (11.49)	0.002	0.030	1494 (12.41)	1518 (12.61)	0.640	0.006
BMI, Mean ± SD	34.92 ± 7.86	34.23 ± 7.86	<0.001	0.089	34.92 ± 7.86	34.70 ± 7.81	0.207	0.027
<30	1334 (11.02)	23426 (15.49)	<0.001	0.132	1330 (11.05)	1292 (10.73)	0.432	0.010
≥30	3233 (26.70)	47728 (31.57)	<0.001	0.107	3221 (26.76)	3020 (25.09)	0.003	0.038
Comorbidities								
Hypertension	3814 (31.50)	59200 (39.15)	<0.001	0.161	3799 (31.56)	3446 (28.62)	<0.001	0.064
Hyperlipidemia	3675 (30.35)	58613 (38.76)	<0.001	0.178	3669 (30.48)	3346 (27.79)	<0.001	0.059
Obesity	1582 (13.06)	24772 (16.38)	<0.001	0.094	1576 (13.09)	1408 (11.70)	0.001	0.042
Chronic kidney disease (CKD)	401 (3.31)	8913 (5.90)	<0.001	0.124	399 (3.31)	349 (2.90)	0.063	0.024
Nicotine dependence	352 (2.91)	5311 (3.51)	<0.001	0.034	349 (2.90)	334 (2.77)	0.560	0.008
Chronic obstructive pulmonary disease	189 (1.56)	2729 (1.81)	0.051	0.019	185 (1.54)	175 (1.45)	0.595	0.007
Alcohol related disorders	71 (0.59)	1020 (0.68)	0.251	0.011	68 (0.57)	66 (0.55)	0.862	0.002
Medications								
Biguanides	2964 (24.48)	47569 (31.46)	<0.001	0.156	2959 (24.58)	2675 (22.22)	<0.001	0.056
Sulfonylureas	1411 (11.65)	18680 (12.35)	0.024	0.022	1409 (11.70)	1268 (10.53)	0.004	0.037
Dipeptidyl peptidase 4 (DPP-4) inhibitors	942 (7.78)	10466 (6.92)	<0.001	0.033	939 (7.80)	907 (7.53)	0.438	0.010
Glucagon-like peptide-1 (GLP-1) analogues	819 (6.76)	18320 (12.12)	<0.001	0.184	819 (6.80)	728 (6.05)	0.017	0.031
Insulins and analogues	1559 (12.87)	23514 (15.55)	<0.001	0.077	1554 (12.91)	1402 (11.65)	0.003	0.038
Thiazolidinediones	252 (2.08)	3236 (2.14)	0.665	0.004	251 (2.09)	231 (1.92)	0.357	0.012
Alpha glucosidase inhibitors	10 (0.08)	149 (0.10)	0.588	0.005	10 (0.08)	10 (0.08)	1.000	<0.001
HMG CoA reductase inhibitors	2370 (19.57)	40338 (26.68)	<0.001	0.169	2366 (19.65)	2098 (17.43)	<0.001	0.057
Aspirin	479 (3.96)	6784 (4.49)	0.006	0.026	478 (3.97)	393 (3.26)	0.003	0.038
Warfarin	44 (0.36)	677 (0.45)	0.178	0.013	44 (0.37)	44 (0.37)	1.000	<0.001
Clopidogrel	16 (0.13)	357 (0.24)	0.021	0.024	16 (0.13)	15 (0.13)	0.857	0.002
Direct factor Xa inhibitors	89 (0.74)	2605 (1.72)	<0.001	0.090	89 (0.74)	81 (0.67)	0.538	0.008
ACE inhibitors, plain	1583 (13.07)	22832 (15.10)	<0.001	0.058	1580 (13.12)	1425 (11.84)	0.003	0.039
Angiotensin II receptor blockers	888 (7.33)	17723 (11.72)	<0.001	0.150	887 (7.37)	798 (6.63)	0.025	0.029
Beta blocking agents	877 (7.24)	15520 (10.26)	<0.001	0.107	875 (7.27)	784 (6.51)	0.021	0.030
Diuretics	1230 (10.16)	21874 (14.47)	<0.001	0.131	1229 (10.21)	1084 (9.00)	0.002	0.041
Laboratory								
eGFR	84.17 ± 27.48	81.68 ± 27.01	<0.001	0.091	84.18 ± 27.49	83.62 ± 25.48	0.326	0.021
HbA1c	8.58 ± 2.19	8.64 ± 1.94	0.039	0.029	8.58 ± 2.19	8.57 ± 2.15	0.789	0.006
Cholesterol in LDL [Mass/volume] in Serum or Plasma	3353 (27.69)	53546 (35.41)	0.466	0.013	3345 (27.79)	3105 (25.79)	0.560	0.015
Cholesterol in LDL [Mass/volume] in Serum or Plasma	91.61 ± 38.35	92.11 ± 38.44	0.466	0.013	91.65 ± 38.37	91.09 ± 39.05	0.560	0.015

SMD: standardized mean difference. If the patient’s count is 1–10, the results indicate a count of 10.

### Primary outcome

**FIGURE 2:**
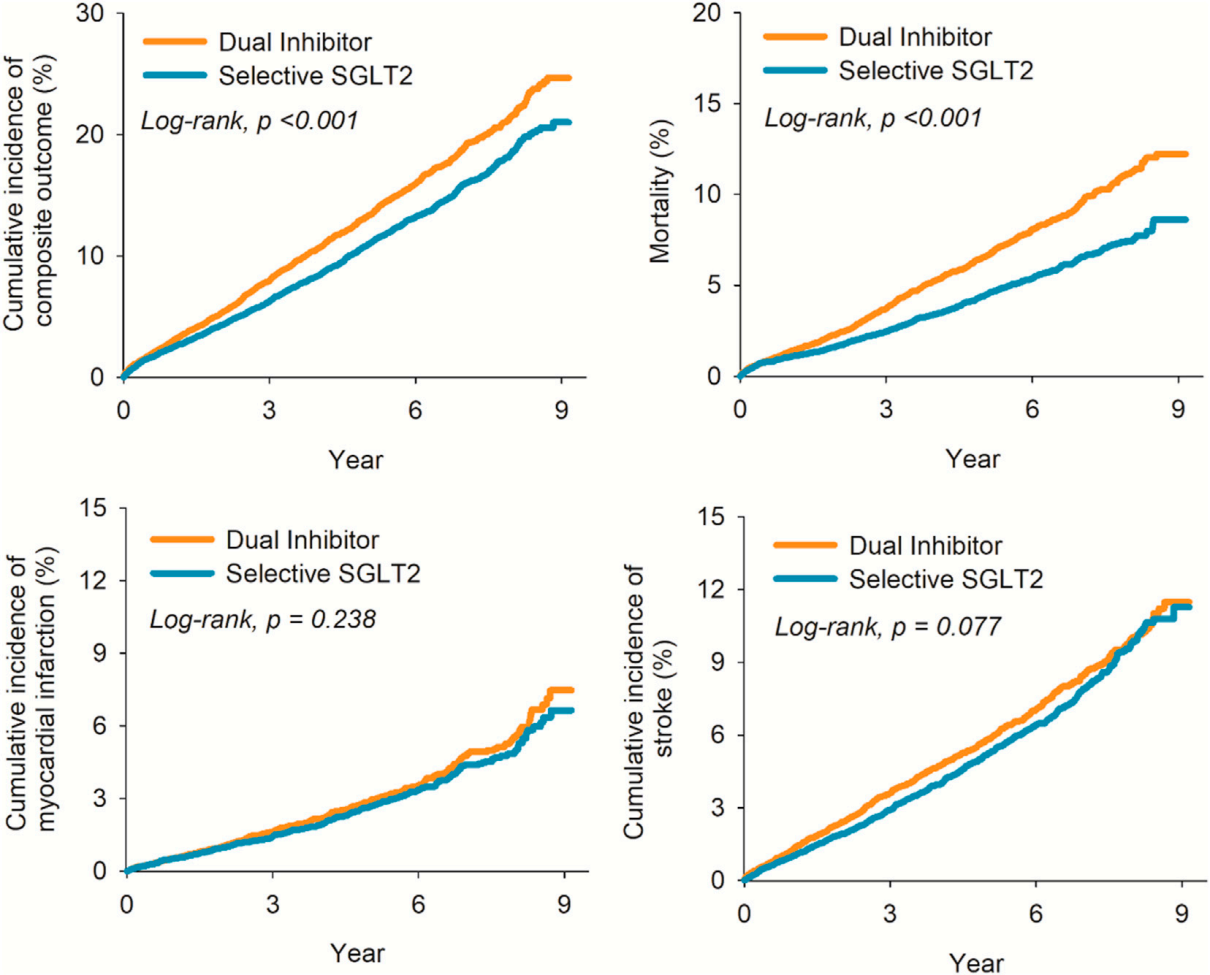
Kaplan-Meier plot for risk of composite outcome (left upper); all-cause mortality (right upper); myocardial infarction (left lower); and stroke (right lower).

During follow-up, 1344 patients in the canagliflozin group and 1206 in the selective SGLT2 group experienced a primary outcome event, while 665 vs. 492 died from any cause (Table 2). The incidence of the composite outcome was significantly higher in patients initiating canagliflozin compared to those on selective SGLT2 inhibitors. The HR for MACE was 1.23 (95% CI 1.14–1.33) in the canagliflozin vs. selective group (p < 0.001). Figure 2 illustrates the Kaplan-Meier curves for the composite outcome, which began to diverge within the first year of therapy and continued to separate over time, favoring the selective SGLT2 group. MI risk did not differ significantly between groups: HR 1.10 (95% CI 0.94–1.29). Similarly, overall stroke (all strokes combined) occurred at a similar rate in both groups: HR 1.11 (0.99–1.24), a non-significant trend toward higher stroke in the dual inhibitor group. When strokes were categorized, ischemic stroke specifically was not significantly different (HR 1.09, 95% CI 0.97–1.23). In contrast, hemorrhagic stroke was notably more frequent in the canagliflozin group: 109 hemorrhagic strokes versus 89 in the selective group (out of 12,039 patients each), corresponding to HR 1.35 (95% CI 1.02–1.79).

**TABLE 2:** Risk of MACE exposed to canagliflozin compared to other selective SGLT2.

	No. of event		
	Canagliflozin N = 12039	Selective SGLT2 N = 12039	HR (95% C.I.)
All	1344	1206	1.23 (1.14–1.33)
Mortality	665	492	1.49 (1.33–1.68)
Myocardial Infarction	306	309	1.10 (0.94–1.29)
Stroke	586	585	1.11 (0.99–1.24)
Hemorrhagic Stroke	109	89	1.35 (1.02–1.79)
Ischemic Stroke	522	531	1.09 (0.97–1.23)

### All-cause mortality

Canagliflozin use was associated with significantly higher all-cause mortality during follow-up. A total of 665 deaths occurred in the canagliflozin group, compared to 492 in the selective SGLT2 group. The HR for all-cause mortality was 1.49 (95% CI 1.33–1.68) in canagliflozin vs. selective users, indicating a 49% relative increase in the risk of death from any cause (p < 0.001). The survival curves separated early and remained apart, with 1-year survival approximately 96.0% in the dual group versus 97.5% in the selective group, and an even larger gap by 3 years. Notably, because we lacked cause-of-death information, we could not determine if cardiovascular mortality specifically was driving this difference.

### Subgroup analyses–MACE composite outcome

We assessed the consistency of the primary composite outcome findings across various patient subgroups (Table 3–6). The elevated MACE risk with canagliflozin was especially pronounced in certain subgroups. In patients younger than 65 years, canagliflozin use was associated with significantly higher risk of MI or stroke (HR 1.14, 95% CI 1.03–1.26), whereas in those ≥65 the HR was 1.02 (0.86–1.20), not reaching significance. Male patients on dual therapy had a higher composite risk (HR 1.24, 95% CI 1.12–1.39), while female patients showed a smaller, non-significant increase (HR ∼1.11, 95% CI 0.99–1.25). When stratified by race, the risk increase was most evident in Asian patients: HR 1.68 (95% CI 1.11–2.54), based on 55 vs. 38 composite events in the canagliflozin vs. selective groups. White patients also showed a significant but more modest risk increase (HR 1.18, 95% CI 1.08–1.29).

**TABLE 3:** Stratification analysis of risk of MACE Composite Outcome exposed to canagliflozin compared to Selective SGLT2.

	Canagliflozin		Selective SGLT2		
	N	No. of event	N	No. of event	HR (95% C.I.)
Age					
<65	8760	771	8760	747	1.14 (1.03–1.26)
≥65	3461	270	3461	291	1.02 (0.86–1.20)
Sex					
Female	5638	613	5638	588	1.11 (0.99–1.25)
Male	5796	685	5796	634	1.24 (1.12–1.39)
Race					
White	7455	964	7455	909	1.18 (1.08–1.29)
Black or African American	1665	171	1665	161	1.15 (0.92–1.42)
Asian	511	55	511	38	1.68 (1.11–2.54)
Body mass index					
<30	1596	227	1596	191	1.33 (1.09–1.61)
≥30	3399	403	3399	369	1.20 (1.04–1.38)
eGFR					
<60	1147	220	1147	200	1.18 (0.97–1.43)
≥60	4332	499	4332	472	1.12 (0.99–1.27)
HbA1c					
<8	3097	351	3097	311	1.21 (1.04–1.41)
≥8	2602	336	2602	311	1.14 (0.98–1.33)

MACE: major advanced cardiovascular events; eGFR: estimated glomerular filtration rate; If the patient’s count is 1–10, the results indicate a count of 10.

**TABLE 4:** Stratification analysis of risk of all-cause mortality exposed to canagliflozin compared to Selective SGLT2.

	Canagliflozin		Selective SGLT2		
	N	No. of event	N	No. of event	HR (95% C.I.)
Age					
<65	8760	352	8760	280	1.38 (1.18–1.62)
≥65	3461	322	3461	244	1.46 (1.24–1.72)
Sex					
Female	5638	280	5638	226	1.32 (1.11–1.57)
Male	5796	352	5796	265	1.52 (1.30–1.78)
Race					
White	7455	478	7455	377	1.41 (1.23–1.61)
Black or African American	1665	74	1665	61	1.30 (0.93–1.82)
Asian	511	19	511	10	2.22 (1.03–4.78)
Body Mass Index					
<30	1596	119	1596	62	2.16 (1.59–2.93)
≥30	3399	192	3399	149	1.42 (1.14–1.75)
eGFR					
<60	1147	125	1147	92	1.44 (1.10–1.89)
≥60	4332	211	4332	157	1.42 (1.16–1.75)
HbA1c					
<8	3097	153	3097	100	1.64 (1.28–2.11)
≥8	2602	157	2602	100	1.67 (1.30–2.14)

eGFR: estimated glomerular filtration rate; If the patient’s count is 1–10, the results indicate a count of 10.

**TABLE 5:** Stratification analysis of risk of Myocardial Infarction exposed to canagliflozin compared to Selective SGLT2.

	Canagliflozin		Selective SGLT2		
	N	No. of event	N	No. of event	HR (95% C.I.)
Age					
<65	8760	190	8760	192	1.11 (0.91–1.35)
≥65	3461	139	3461	122	1.26 (0.99–1.61)
Sex					
Female	5638	147	5638	155	1.02 (0.82–1.28)
Male	5796	165	5796	155	1.24 (0.99–1.54)
Race					
White	7455	239	7455	250	1.07 (0.89–1.28)
Black or African American	1665	44	1665	43	1.10 (0.72–1.67)
Asian	511	13	511	10	1.66 (0.71–3.88)
Body Mass Index					
<30	1596	51	1596	51	1.12 (0.76–1.65)
≥30	3399	92	3399	119	0.85 (0.65–1.11)
eGFR					
<60	1147	55	1147	61	0.97 (0.67–1.39)
≥60	4332	125	4332	138	0.96 (0.76–1.23)
HbA1c					
<8	3097	86	3097	91	1.02 (0.76–1.37)
≥8	2602	83	2602	103	0.85 (0.64–1.13)

eGFR: estimated glomerular filtration rate; If the patient’s count is 1–10, the results indicate a count of 10.

**TABLE 6:** Stratification analysis of risk of Stroke exposed to canagliflozin compared to Selective SGLT2.

	Canagliflozin		Selective SGLT2		
	N	No. of event	N	No. of event	HR (95% C.I.)
Age					
<65	8760	330	8760	372	0.98 (0.85–1.14)
≥65	3461	270	3461	291	1.02 (0.86–1.20)
Sex					
Female	5638	269	5638	293	0.98 (0.83–1.16)
Male	5796	279	5796	314	1.02 (0.87–1.20)
Race					
White	7455	391	7455	437	1.00 (0.87–1.14)
Black or African American	1665	72	1665	75	1.03 (0.75–1.42)
Asian	511	32	511	25	1.47 (0.87–2.48)
Body Mass Index					
<30	1596	90	1596	98	1.02 (0.77–1.36)
≥30	3399	179	3399	175	1.13 (0.92–1.39)
eGFR					
<60	1147	86	1147	90	1.02 (0.76–1.38)
≥60	4332	228	4332	246	0.99 (0.82–1.18)
HbA1c					
<8	3097	163	3097	164	1.07 (0.86–1.33)
≥8	2602	145	2602	156	0.98 (0.78–1.23)

eGFR: estimated glomerular filtration rate; If the patient’s count is 1–10, the results indicate a count of 10.

### Subgroup analyses–all cause mortality

In analyses of all-cause mortality across subgroups (Table 4), the pattern was even more uniformly in favor of selective SGLT2 inhibitors. Every subgroup examined showed higher mortality risk with canagliflozin use. For example, in patients <65: HR 1.38 (95% CI 1.18–1.62); in ≥65: HR 1.46 (1.24–1.72). In female patients: HR 1.32 (1.11–1.57); in male patients: HR 1.52 (1.30–1.78). White patients had HR 1.41 (1.23–1.61). Notably, even among patients with poorer glycemic control (HbA1c ≥ 8%), mortality was higher on canagliflozin (HR 1.67), and similarly in those with HbA1c <8% (HR 1.64). These consistent findings across subpopulations bolster the primary result that canagliflozin was associated with worse survival than selective SGLT2 inhibition in our cohort.

### Subgroup analysis–MI and stroke

The risk of MI and stroke remained neutral across all examined subgroups, with no statistically significant differences between canagliflozin and selective SGLT2 inhibitors. This pattern remained consistent age, sex, race, BMI, eGFR and baseline HbA1c.

## Discussion

In this large real-world analysis of adults with type 2 diabetes, initiation of canagliflozin was associated with a higher risk of MACE and all-cause mortality compared to initiation of a selective SGLT2 inhibitor. Specifically, canagliflozin use was linked to a 23% higher hazard of composite MACE and nearly 50% higher hazard of all-cause mortality. The risk of hemorrhagic stroke was significantly elevated with canagliflozin, even though overall stroke and MI risks were similar between groups. These findings were consistent across most patient subgroups and were robust in the propensity-matched cohort. To our knowledge, this represents one of the first routine-practice comparisons of less selective versus more selective SGLT2 inhibition, and the findings are best regarded as exploratory observations that highlight areas for further investigation.

Our observations appear different from what has been reported in randomized clinical trials of SGLT2 inhibitors. In the CANVAS Program and CREDENCE trial, (Neal et al., 2017; Perkovic et al., 2019), canagliflozin significantly reduced MACE compared with placebo. Empagliflozin also reduced MACE and cardiovascular mortality in EMPA-REG OUTCOME, (Zinman et al., 2015), whereas dapagliflozin (DECLARE–TIMI 58) (Wiviott et al., 2019) and ertugliflozin (VERTIS CV) (Cannon et al., 2020) were neutral for MACE, though both demonstrated consistent benefits for heart failure and renal outcomes. Dual SGLT1/2 inhibition with sotagliflozin (SOLOIST-WHF and SCORED) likewise improved cardiovascular outcomes in high-risk populations. (Aggarwal et al., 2025; Bhatt et al., 2021b). Taken together, these findings suggest that both selective and less selective SGLT inhibitors confer important cardiometabolic benefits compared with no SGLT therapy, though their effects on atherosclerotic outcomes appear heterogeneous. In this context, our real-world analysis may reflect differences in patient populations, outcome definitions, or prescribing patterns rather than intrinsic harm from canagliflozin.

How, then, do we reconcile those findings with our observation that canagliflozin were associated with less favorable outcomes than selective SGLT2 inhibitors in a real-world comparison? Several considerations and potential mechanisms may explain this discrepancy. First, differences in patient populations and prescribing patterns could contribute. Our study focused on patients without established cardiovascular disease at baseline (a primary prevention cohort), whereas many SGLT2 inhibitor trials enrolled secondary prevention populations. In primary prevention settings, the absolute cardiovascular benefit of any SGLT2 inhibitor is smaller, and thus any differences or risks might become more apparent. It is possible that clinicians tended to prescribe canagliflozin to slightly different patient profiles than those given empagliflozin or dapagliflozin, even though we matched on measured covariates. Unmeasured confounders, such as socioeconomic factors, medication adherence, or untreated risk factors might have been imbalanced. For instance, if canagliflozin was more often used in patients who had longer diabetes duration or were intolerant of other drugs, those patients might inherently have had higher risk. We tried to account for many factors, but residual confounding is an inherent limitation of observational studies. These findings should be regarded as exploratory, given the limitations of observational data.

Second, drug-specific effects may also be considered. In our data, canagliflozin was the predominant dual SGLT1/2 agent, as sotagliflozin had limited clinical use during the study period. Canagliflozin’s clinical trial history has included observations such as a higher incidence of amputations and an early imbalance in stroke in the CANVAS Program, though later analyses did not confirm an increased stroke risk. (Neal et al., 2017). It is possible that SGLT1 inhibition could influence physiology, for example, by altering glucose handling in the gut, contributing to osmotic effects, diarrhea, or volume depletion, which in turn might affect cerebral perfusion in susceptible patients. (Tsimihodimos et al., 2018). SGLT1 inhibition could also impact the gut microbiome and metabolic milieu in complex ways. (Lehmann and Hornby, 2016). At the same time, it is important to note that Mendelian randomization analyses suggest reduced SGLT1 activity is associated with lower mortality and improved cardiometabolic profiles, and that randomized trials of both canagliflozin and sotagliflozin have demonstrated cardiovascular benefits. (Seidelmann et al., 2018; Katzmann et al., 2021). Thus, while mechanistic hypotheses are worth exploring, the available evidence overall does not support the conclusion that SGLT1 inhibition is harmful; (Sayour et al., 2024); rather, our observations should be seen as preliminary and requiring further validation.

The observation of a higher rate of hemorrhagic stroke with canagliflozin is intriguing but should be interpreted with great caution. None of the large SGLT2 inhibitor trials reported a significant increase in hemorrhagic stroke, (Ong et al., 2022), and the absolute number of such events in our study was small. This pattern could therefore reflect chance variation, coding differences, or residual confounding rather than a true drug effect. One speculative explanation is that greater blood pressure reductions or volume contraction with less selective agents might predispose vulnerable individuals, although such mechanisms remain unproven. In contrast, a recent meta-analysis of randomized trials found that less selective SGLT2 inhibitors were actually associated with a lower overall risk of stroke. (Sayour et al., 2024). Taken together, our findings should be regarded as exploratory observations that highlight the importance of further research, ideally with careful adjudication of stroke subtypes, before drawing firm conclusions.

This study has several limitations that are important for interpreting the results. First, as an observational study using EHR data, it is susceptible to residual confounding and bias. We addressed many known confounders through matching and adjustments, but unmeasured factors (such as dietary habits, over-the-counter medication use, or provider preference) could influence both the choice of selective SGLT2 vs. canagliflozin and the risk of outcomes. Second, our outcome definitions relied on diagnostic coding, which may misclassify events. However, we expect any misclassification to be non-differential between groups, which would bias toward null findings rather than create false positive differences. Third, we lacked data on cause of death, distinguishing cardiovascular vs. non-cardiovascular mortality could provide insight. Fourth, our cohort did not include patients with established cardiovascular disease, so the findings may not generalize to a secondary prevention population. Finally, we excluded sotagliflozin entirely due to minimal data, so these findings reflect canagliflozin use specifically and cannot be extrapolated to other dual inhibitors.

Despite these limitations, our study also has notable strengths. It leverages a very large and diverse patient population from real-world clinical practice, increasing the generalizability of the findings. The use of rigorous matching and a breadth of covariate adjustments lends credibility to the observed associations. We were able to examine multiple clinically relevant outcomes, including mortality and stroke subtypes, which have not been directly compared between canagliflozin and selective SGLT inhibitors before. The consistency of the mortality finding across all subgroups suggests that this is a robust signal.

In summary, our observational analysis suggested higher event rates with canagliflozin compared to selective SGLT2 inhibitors in patients with T2DM without established cardiovascular disease. These findings should be interpreted as exploratory and hypothesis-generating, particularly given contrasts with randomized trial and genetic evidence that support benefits of both selective and less selective agents. Rather than indicating harm, our observations highlight the need for further investigation, ideally through head-to-head randomized studies or quasi-experimental approaches, to better understand potential differences among SGLT2 inhibitors in real-world practice.

## Data Availability

This population-based study obtained data from the TrinetX platform (accessible at https://trinetx.com/), for which third-party restrictions apply to the availability of this data. The data were used under license for this study with restrictions that do not allow for data to be redistributed or made publicly available. To gain access to the data, a request can be made to TriNetX (join@trinetx.com), but costs might be incurred, and a data-sharing agreement would be necessary.
