# Supplementary material for: Comparative cardiovascular risks of canagliflozin and selective SGLT2 inhibitors in type 2 diabetes
Source: Front Pharmacol. 2025 Oct 20;16:1686851. doi: 10.3389/fphar.2025.1686851 (PMC12580561; doi:10.3389/fphar.2025.1686851)
Supplement: Supplementary file 1 [file Table1.docx]

**Supplemental table 1**. Detailed coding of this study

| **Outcomes** | **Code** |
| --- | --- |
| Myocardial infarction | ICD-10: I32. I22 |
| Stroke |  |
| Hemorrhagic stroke | ICD-10: I60, I61, I62 |
| Ischemic stroke | ICD-10: I63, I65, I66 |
| All-cause mortality | TNX: mortality |
|  |  |
| **Cohort selection** |  |
| Dual inhibitor SGLT1/2 |  |
| Canagliflozin | RxNorm: 1373458 |
| Sotagliflozin | RxNorm: 2638675 |
|  |  |
| Selective SGLT2 |  |
| Empagliflozin | RxNorm: 1545653 |
| Dapagliflozin | RxNorm: 1488564 |
| Ertugliflozin | RxNorm: 1992672 |
|  |  |
| Exclusion |  |
| Transient cerebral ischemic attacks and related syndromes | ICD-10: G45 |
| Ischemic heart diseases | ICD-10: I20-I25 |
| Cerebrovascular diseases | ICD-10: I60-I69 |
| Atherosclerosis | ICD-10: I70 |
| Other peripheral vascular diseases | ICD-10: I73 |
| Type 1 diabetes mellitus | ICD-10: E10 |
| Diabetes mellitus in pregnancy, childbirth, and the puerperium | ICD-10: O24 |
| End stage renal disease | ICD-10: N18.6 |
| Dependence on renal dialysis | ICD-10: Z99.2 |
|  |  |
| **Comorbidities** |  |
| Essential (primary) hypertension | ICD-10: I10 |
| Disorders of lipoprotein metabolism and other lipidemias | ICD-10: E78 |
| Overweight and obesity | ICD-10: E66 |
| Chronic kidney disease (CKD) | ICD-10: N18 |
| Nicotine dependence | ICD-10: F17 |
| Other chronic obstructive pulmonary disease | ICD-10: J44 |
| Alcohol related disorders | ICD-10: F10 |
| **Medications** |  |
| Biguanides | ATC: A10BA |
| Sulfonylureas | ATC: A10BB |
| Dipeptidyl peptidase 4 (DPP-4) inhibitors | ATC: A10BH |
| Glucagon-like peptide-1 (GLP-1) analogues | ATC: A10BJ |
| INSULINS AND ANALOGUES | ATC: A10A |
| Thiazolidinediones | ATC: A10BG |
| Alpha glucosidase inhibitors | ATC: A10BF |
| HMG CoA reductase inhibitors | ATC: C10AA |
| Aspirin | RxNorm: 1191 |
| Warfarin | RxNorm: 11289 |
| Clopidogrel | RxNorm: 32968 |
| Direct factor Xa inhibitors | ATC: B01AF |
| ACE inhibitors, plain | ATC: C09AA |
| Angiotensin II receptor blockers (ARBs), plain | ATC: C09CA |
| BETA BLOCKING AGENTS | ATC: C07A |
| DIURETICS | ATC: C03 |
| **Laboratory** |  |
| Glomerular filtration rate/1.73 sq M.predicted [Volume Rate/Area] in Serum,  Plasma or Blood by Creatinine-based formula (MDRD) | TNX: 8001 |
| Hemoglobin A1c/Hemoglobin.total in Blood | TNX: 9037 |
| Cholesterol in LDL [Mass/volume] in Serum or Plasma | TNX: 9002 |

ICD-10-CM: International Classification of Diseases, Tenth Revision, Clinical Modification.

RxNorm: Medical prescription normalized Medical prescription.

ATC: Anatomical Therapeutic Chemical.

TNX: TrinetX curated.
